# Anti-infectious and anti-inflammatory effect of amniopatch in the treatment of spontaneous previable rupture of membranes

**DOI:** 10.1007/s00404-024-07399-0

**Published:** 2024-04-20

**Authors:** Martin Alföldi, Vladimír Ferianec

**Affiliations:** 2nd Department of Gynaecology and Obstetrics, Faculty of Medicine Comenius University (FMCU) and University Hospital (UH) Bratislava, 6 Ružinovská Str, 82606 Bratislava, Slovakia

**Keywords:** Anti-infectious effect, Anti-inflammatory effect, Non-coagulant components, Amniopatch, Treatment, Spontaneous previable rupture of membranes, Potential, Method

## Abstract

Spontaneous previable rupture of membranes complicates approximately 0.4–0.7% of pregnancies and is associated with severe maternal and neonatal morbidity and mortality. Intra-amniotic inflammation is present in up to 94.4% of cases, most often caused by a bacterial infection. In comparison, the effectiveness of antibiotic therapy in its eradication reaches less than 17%. Inflammatory activity in the amniotic cavity disrupts the physiological development of the fetus with an increase in maternal, fetal, and neonatal inflammatory morbidity through the development of fetal inflammatory response syndrome, maternal chorioamnionitis, and neonatal sepsis. Amniopatch is an invasive therapeutic technique based on intra-amniotic administration of maternal hemoderivates in the form of thromboconcentrate and plasma cryoprecipitate to provide the temporary closure of the fetal membranes defect and secondary restitution of normohydramnios with correction of pressure–volume ratios. The supposed basis of this physical–mechanical action is the aggregation of coagulant components of amniopatch in the area of the defect with the formation of a valve cap. The background for the formulation of the hypothesis on the potential anti-infectious and anti-inflammatory action of non-coagulant components of amniopatch involved: i) clinical–academic and publishing outputs of the authors based on their many years’ experience with amniopatch application in the treatment of spontaneous previable rupture of membranes (2008–2019), ii) the documented absence of clinically manifested chorioamnionitis in patients treated this way with a simultaneously reduced incidence of neonatal respiratory distress syndrome compared to expectant management (tocolysis, corticotherapy, antibiotic therapy). The non-coagulant components of plasma cryoprecipitate include mainly naturally occurring isohemagglutinins, albumin, and soluble plasma fibrinogen. Although these components of the amniopatch have not been attributed a significant therapeutic role, the authors assume that due to their opsonizing and aggregative properties, they can significantly participate in optimizing the intrauterine environment through the reduction in bacterial and cytokine charge in the amniotic fluid. The authors think these facts constitute a vital stimulus to future research–academic activity and, at the same time, an idea for reconsidering the therapeutic role of amniopatch as a tool for improving perinatal results of spontaneous previable ruptures of membranes.

## Introduction

Spontaneous previable premature rupture of membranes (sPPROM) is defined as disruption of the integrity of the chorioamniotic sac in a previable period with no association with the invasive intrauterine procedure. The progressive reduction of amniotic fluid volume, contamination of the amniotic fluid by the cervical-vaginal-perineal microbiome, and organ immaturity of the fetus are the essential determinants of the highly unfavourable prognosis for pregnancy.

Perinatal mortality in such compromised pregnancies reaches 60%, with one-third of fetuses dying in utero [1].

Almost without exception, liveborn neonates are burdened with potentially lethal, disabling complications resulting from extreme prematurity (pulmonary hypoplasia [PH], intraventricular haemorrhage [IVH], periventricular leukomalacia [PVL], respiratory distress syndrome [RDS], necrotizing enterocolitis [NEC] and retinopathy of prematurity [ROP]), intra-amniotic inflammation (IAI), sepsis, pneumonia, meningitis), and depletion of the amniotic fluid with fetal movement restriction and forced fetal habit (fetal compression syndrome [FCS]). No less serious is the high infectious morbidity of the mothers affected by clinically manifested chorioamnionitis (ChA) [1–3].

PH presents the crucial factor limiting the effective cardiorespiratory adaptation of the sPPROM newborns. The condition is defined by a reduction in the number of airways, alveoli and lung cells. This is caused mainly by an alteration in the amount of lung fluid during the canalicular stage of fetal lung development (from 17–24 gestational week [gw]). The incidence of sPPROM associated PH ranges from 13 to 28%. The most important independent risk factors associated with sPPROM are represented by (1) early gestational age at sPPROM (50–60% risk of PH development when sPPROM occurs at 20 gw) and (2) low residual amniotic fluid volume (amniotic fluid index [AFI] <5 cm or deepest vertical pocket [DVP] <2 cm) at the time of the diagnosis. The mortality rate of neonates suffering from this condition ranges from 50 to 95%. The gold standard for diagnosing PH is lung weight by autopsy, which cannot be used in the antenatal period [5, 6].

Possible antenatal diagnostic tests for PH are (1) amniotic fluid volume measurement (AFI, DVP), (2) fetal breathing movements detection, (3) sonoanthropometry (length, volume) and doplerometry of fetal lungs and pulmonary vessels. Predictive accuracy is generally poor and may be improved by combining abovementioned tests [8].

Clinically manifest or asymptomatic IAI and ChA complicate about 95% of sPPROM cases, with more than 50% of symptomatic forms presenting within the first 7 days after rupture detection (maximum clinical occurrence between Day 2 and 5). After the first 7 days of the latency period (LP), the incidence of clinically manifest IAIs dramatically falls, primarily due to their chronic, subclinical nature [9, 10].

The most common cause of IAI is the bacterial invasion of the amniotic cavity (present in more than 94.4% of cases). The bacteria can cross the intact fetal membranes (FM), with secondary colonization of the regional surfaces (amniotic membranes, umbilical cord, fetal skin) and inflammation inducement. In the setting of IAI, the amniotic cavity contains two phenotypically different types of bacteria: (1) bacteria “freely” floating in the amniotic fluid (“free-floating bacteria”) and (2) biofilm-forming bacteria (“adherent bacteria”). The abovementioned subpopulations play a distinct role in the pathophysiology of sPPROM because of their different susceptibility to antimicrobial treatment [11].

The IAI can also be induced by high levels of endogenous molecules (nucleic acids, mitochondrial proteins, potassium ions, glucose, and non-enzymatically glycated proteins) released from apoptotic cells. This phenomenon is much rarer (7% of cases of sPPROM) and was described in the process of premature FM senescence (pathologically accelerated apoptosis of amniocytes) [12, 13].

Subclinical IAI (microbially induced or sterile) is the most common pathophysiological pathway for FM weakening and rupture. Another important fact is that intra-amniotic-inflammatory activity can lead to fetal inflammatory response syndrome (FIRS), which interferes with the physiological development of the fetus and functional organ maturation. Thus, it is associated with shortening the LP from detection of sPPROM to childbirth (higher incidence of childbirth up to 48 and 72 h from PPROM detection compared to the absence of FIRS) and increased rates of neonatal morbidity in the following categories: 1. early neonatal sepsis, 2. bronchopulmonary dysplasia (BPD), IVH, PVL and RDS compared to its absence. Despite the multi-organ nature of the fetal disability, FIRS is predominantly associated with fetal brain damage [14, 15].

The presence of IAI/ChA in cases of mid-trimester sPPROM poorly correlates with maternal clinical (fever, tachycardia, purulent discharge, uterine tenderness [Gibbs criteria]) and laboratory signs (leukocytosis, elevated levels of proteins of the acute phase of inflammation) of inflammation. The reason for this phenomenon is the relative separation of the amniotic cavity from the maternal bloodstream compartment; therefore, the overt signs of maternal systemic inflammatory response syndrome (SIRS) present a late form of manifestation with a high probability of fetal damage. The diagnosis of IAI/ChA is usually made by examination of amniotic fluid for IL-6 concentration and the presence of bacteria (cultivation of amniotic fluid samples) or their nucleic acids (nucleic acid amplification test [NAAT]). The summary of different cut-off values based on the examined material and method used is presented in Table 1 [16, 17].

**Table 1:** The summary of clinical use of IL-6 in diagnosis of IAI/ChA and FIRS—adapted from [16, 17]

Diagnosis	Examined material	Method used	Cut-off (IL-6 pg/ml)	Sensitivity (%)	Specificity (%)	NPV (%)	PPV (%)
IAI/ChA	Amniotic fluid	ELISA	2600 <	100	50	100	70.6
		ECLIA	3000 <	88	99	96	97
		POC test	745 <	50	95	81	82
FIRS	Fetal blood	ELISA	11 <	100	30.3	100	30.3

*IAI* intra-amniotic inflammation, *ChA* chorioamnionitis, *IL-6* interleukin 6, *FIRS* fetal inflammatory response syndrome, *NPV* negative predictive value, *PPV* positive predictive value, *ELISA* enzyme-linked immunosorbent assay, *ECLIA* electrochemiluminescence immunoessay, *POC* point of care test

Based on the presence or absence of IAI and bacteria in the amniotic fluid of sPPROM patients, those can be divided into four different phenotypic categories. We believe this fact has a crucial effect on the effectiveness of therapeutic strategies and pregnancy prognosis. The characteristics of specific phenotypes are summarized in Table 2 [18].

**Table 2:** Phenotypic sPPROM profile of amniotic and fetal compartment depending on the presence or absence of intra-amniotic inflammation and bacterial colonization—adapted from [18]

Phenotypic sPPROM profile—amniotic compartment—amniotic fluid		
sPPROM phenotype	IL-6 concentration in amniotic fluid (pg/ml)	The presence of bacteria in the amniotic fluid (culture, PCR)
Intra-amniotic inflammation	ECLIA ≥ 3000 ELISA ≥ 2600 POC test ≥ 745	The absence of bacteria in the amniotic fluid
Intra-amniotic infection	ECLIA ≥ 3000 ELISA ≥ 2600 POC test ≥ 745	The presence of bacteria in the amniotic fluid
Microbial invasion of the amniotic cavity	ECLIA ≤ 3000 ELISA ≤ 2600 POC test ≤ 745	The presence of bacteria in the amniotic fluid
“Negative” amniotic fluid	ECLIA ≤ 3000 ELISA ≤ 2600 POC test ≤ 745	The absence of bacteria in the amniotic fluid
Phenotypic sPPROM profile—fetal compartment—fetal blood		
FIRS (microorganisms-related induced form)	ELISA ≥ 1100	The presence of bacteria in the fetal tissues
FIRS (sterile form)	ELISA ≥ 1100	The absence of bacteria in the fetal tissues
The absence of FIRS	ELISA ≤ 1100	The absence of bacteria in the fetal tissues

*IL-6* interleukin 6, *sPPROM* spontaneous previable rupture of membranes, *PCR* polymerase chain reaction, *ECLIA* electrochemiluminescence method, *ELISA* enzyme immunoassay, *POC* point of care test, *FIRS* fetal inflammatory response syndrome

Currently, there are limited options available for primary prevention of sPPROM. The only possible action to reduce the occurrence of sPPROM in the general population is the preconception eradication of vulvovaginal dysmicrobia and extragenital infectious foci. The whole population screening for such diagnoses is inappropriate because of their low prevalence and high financial necessity. The only rational approach is high-risk population preconception screening (patients with a history of sPPROM or other clinical forms of preterm labour [PTL]) with subsequent treatment in cases of positivity [19, 20].

The secondary prevention of sPPROM is complicated and cannot be applied in current clinical practice. The reason for this is the long asymptomatic period of subclinical IAI, which usually precedes the clinical manifestation of sPPROM for several weeks. There has been some research interested in the early detection of possible IAI based on continuous monitoring of maternal physiological functions (body temperature, pulse rate, breath rate) and laboratory examination (levels of C-reactive protein [CRP], leukocytes). However, the results and clinical feasibility still need to be more conclusive regarding the sPPROM topic [21]. During this asymptomatic “window”, rising levels of proinflammatory cytokines in the amniotic cavity induce proteolytic enzymes, which in turn weaken FM, leading to their subsequent rupture [12, 14]. The noninvasive screening tests for the detection of subclinical IAI from maternal blood or other bodily fluids will be necessary in future clinical practice to select the population with a high risk of IAI presence for further invasive diagnostic procedures (amniocentesis [AMC] and fetal blood sampling [FBS]). Invasive testing of the general low-risk population is inadmissible because of the risk of pregnancy loss associated with the procedure and its low cost-effectiveness profile.

FCS is a severe sPPROM complication arising from asymmetric intrauterine pressure being exerted on the fetus in the condition of reduced or absent amniotic fluid volume. The fixed fetal habitus and restriction of fetal movements result in limb and joint position deformities and craniofacial deformities of varying severity. The mean frequency of FCS in cases of sPPROM is 7%. The duration of LP and the extent of amniotic fluid depletion are the most important independent predictors of severity. The gw of sPPROM is not a significant determinant because of ongoing axial skeleton development [22].

Placental abruption is a frequent complication of sPPROM, complicating around 44% of such pregnancies in comparison with the 0.8% risk of the general obstetric population. The most important risk factors are: (1) low gw at the time of sPPROM and (2) the presence of fresh vaginal bleeding before sPPROM detection [23, 24].

Cord prolapse is a complete or partial protrusion of the umbilical cord through the defect in the FM between the presenting fetal part and the structures of the bony pelvis. It presents an obstetric emergency with complete obstruction of the umbilical vessels. The incidence of cord prolapse in cases of sPPROM is around 2% with vertex presentation but rises to 11% with breech presentation or transverse fetal lie [25].

Other neonatal morbidities are predominantly caused by extreme prematurity and have been listed in the text above. They have a multi-organ nature and various severity levels (IVH, PVL, ROP, RDS, and NEC) [9].

The list of maternal sPPROM-associated complications includes (1) retained placenta, and (2) endometritis. Postpartum/post-abortion endometritis occurs in about 13% of cases, with about 0.8% of cases progressing to a septic state. The likelihood of clinical presentation rises with the prolongation of the latency interval (interval from sPPROM detection to delivery or abortion). Retention of the placenta with the subsequent necessity of instrumental revision of the uterine cavity occurs in 9**–**18% of cases. The risk of complications rises with the lower sPPROM gw [12].

## Review

sPPROM complicates 0.4**–**0.7% of pregnancies. IAI is observed in 94.4% of cases and is primarily attributed to bacteria. The eradication effectiveness of standard antibiotic therapy reaches only 17%. The pernicious nature of the condition stems from its limited therapeutic options [1].

sPPROM has very limited options for therapeutic influence and is often associated with therapeutic nihilism in the literature. That fact results from the limited, clinically insignificant tendency of the FM to spontaneous healing and the relative absence of therapeutic agents intensifying the activity referred to, which ultimately does not allow the causal treatment of the diagnosis [26, 27]. Due to the unfavourable prognostic profile of pregnancy, certain patients opt for artificial interruption, which is deemed an acceptable therapeutic alternative in context-sensitive relationships. In the case of a request from both parents to continue the pregnancy, opinions about its further management are incoherent and predominantly depend on the gestational week of PPROM [28].

The spectrum of available therapeutic modalities includes two philosophically heterogeneous approaches (1) expectant management with the application of intrauterine pharmacotherapy to improve postnatal organ adaptation of the fetus (tocolysis, corticotherapy, antibiotic therapy), and (2) active management with the invasive, intra-amniotic application of biocompatible substances to close the defect of FM with secondary restitution of normohydramnios and pressure–volume ratios in the amniotic cavity.

The ultimate “therapeutic” option can be the delivery (termination) of the affected pregnancy. As we already mentioned, this can be done by parental request after careful consideration or in the presence of (1) intrauterine fetal demise (IUFD), (2) spontaneous onset of labour, (3) evidence of maternal or fetal infection, and (4) obstetric emergency requiring immediate delivery (cord prolapse, placental abruption, severe, resistant preeclampsia).

The therapeutic effect of standardly applied sPPROM drug therapy was not sufficiently documented in the previability and extreme prematurity zone, which was also considered in the sixth update of the joint recommendation of the American College of Obstetricians and Gynecologists (ACOG) and the Society for Maternal–Fetal Medicine (SMFM) (October 2017) for the treatment of premature birth syndrome in the previable period (Table 3) [29].

**Table 3:** 6th Common Expert Guidance of the American College of Obstetricians and Gynecologists (ACOG) and the Society for Maternal–Fetal Medicine (SMFM) for the treatment of pre- and periviable periods (October 2017)—adapted from [29]

Particular gestational weeks in the periviable period					
Therapeutic modality	20_0/7_–21_6/7_ gw	22_0/7_–22_6/7_ gw	23_0/7_–23_6/7_ gw	24_0/7_–24_6/7_ gw	25_0/7_–25_6/7_ gw
Antenatal corticosteroids	Not recommended	Not recommended	Possible after a thorough consideration	Recommended	Recommended
Magnesium sulfate for neuroprotection	Not recommended	Not recommended	Possible after a thorough consideration	Recommended	Recommended
Tocolysis	Not recommended	Not recommended	Possible after a thorough consideration	Recommended	Recommended
Antibiotics for prophylaxis	Possible after a thorough consideration	Possible after a thorough consideration	Possible after a thorough consideration	Recommended	Recommended

*gw* gestational week

Active management includes several techniques that have been designed for the purpose of artificial closure of a defect in the amniotic sac or replacement of amniotic fluid volume, referred to in the Anglo-Saxon literature as the so-called “resealing techniques” or “amniotic fluid reparation techniques” (AFRT) [30].

Amnioinfusion (AI) is a technique based on the sterile, transabdominal intra-amniotic application of amniotic fluid-like solutions (warmed Ringer lactate or saline solution). It was first introduced in the 1960s as a possible means of pregnancy termination and labour induction in cases of IUFD. Nowadays, its dominant clinical use is in correcting abnormal intrapartum cardiotocography (CTG) patterns caused by umbilical cord compression (repeated variable decelerations). In cases of sPPROM-associated morbidity and mortality, serial transabdominal AI aims to enhance the depleted amniotic fluid volume, reduce the occurrence of PH, prolong the LP, and improve the perinatal outcome [31, 32].

Based on the published literature, there is considerable controversy over the role of serial transabdominal AI in the treatment protocol for sPPROM. The generally prevailing opinion is that serial transabdominal AI does not improve sPPROM-associated mortality and morbidity, mainly because of the short maintenance rates of amniotic fluid. The average maintenance rate of 48 h after administration is successfully achieved in 24–31% of pregnancies. Although some reports describe successful prolongation of the LP, there has been no change in the perinatal outcomes. The promising alternative being currently discussed is the continuous long-term AI. The procedure is also called the “flush-out” technique, emphasizing its similarity with irrigation and debridement of infectious foci. The principle of the technique is based on the restitution of amniotic fluid volume and the decrease in local concentrations of bacteria and cytokines through the continuous flow of “amniotic fluid-like” solutions via the amniotic cavity [2, 34].

The exact role of continuous AI in clinical practice awaits more RCTs. The papers from Japan (2020) comparing the effect of continuous AI on perinatal outcome in cases of sPPROM report no statistically significant differences compared to expectant management. A German group led by Tchirikov is performing a registered clinical trial comparing the effect of continuous AI on expectant management in terms of perinatal outcome. The published protocol of the study (2022) contains 31 patients with sPPROM in both arms of the study. The working group emphasizes the importance of the similarity of the solutions used with amniotic fluid because of their effect on genetic and metabolic programming and fetal cellular membrane function. The high concentrations of some ions (sodium, potassium, chloride) can increase fetal and neonatal morbidity and mortality [35, 36].

One of the “pioneer” methods of active management of sPPROM is represented by amniopatch (AP), described for the first time by R. A. Quintero in 1996 in connection with the treatment of previable rupture of membranes complicating intrauterine operational procedures [38]. The principle of the technique is the sterile, transabdominal, intra-amniotic application of maternal hemoderivates (platelets and plasma cryoprecipitate) to form a temporary closure of the FM defect [39]. The therapeutic protocol for its application includes a two-step scheme of separating maternal hemoderivates, their manufacturing and subsequent intra-amniotic administration [1] (Table 4).

**Table 4:** Protocol of preparation and application of amniopatch used by the authors at the 2nd Clinic of Gynaecology and Obstetrics FMCU and UH Bratislava [1]

Application protocol		
Step	1	2
Procedure carried out	Separation of maternal hemoderivates using the HAE-MONETICS MCS plus® separator (LDP protocol card, set number 994 CFE, [Haemonetics Corp., Braintree, MA]) for two 45-min cycles (autotransfusion protocol)	Intra-amniotic transabdominal application of hemoderivates using a 22G puncture needle under continuous USG guiding with immediate USG monitoring of the fetus in the post-procedure period and repeated USG follow-up on postoperative day 1

*G* gauge, *USG* ultrasound

The assessment of the success rate of this technique depends on the criteria applied and has undergone considerable development since its clinical introduction. In the 1990s, the complete cessation of amniotic fluid leakage with pregnancy prolongation to the due date was considered a solitary criterion for success. Nowadays, the focus is mainly on the perinatal mortality and morbidity rate of pregnancies treated this way. This is documented by the statement of the authors of a 2022 publication from the Mayo Rochester Clinic (focused on treatment with sPPROM AP published in *The Journal of Maternal–Fetal & Neonatal Medicine* entitled *Interventional resealing of preterm premature rupture of the membranes: a systematic review and meta-analysis*, where in the discussion part they state the following conclusion: “*Maternal, fetal, and neonatal outcomes are perhaps the most important clinical consideration when evaluating the suitability of amniopatch for PPROM in all forms*” [40]. The authors of this paper came to a similar conclusion in an article of May 2022 published in *Bratislava Medical Journal* entitled *Is amniopatch an effective treatment for spontaneous previable premature rupture of membranes? Analysis of perinatal outcomes*, where, in conclusion, they report a reduced incidence of RDS and ROP with a simultaneously lower incidence of clinically manifested ChA in cases of sPPROM treated with AP application [1, 41]. The summary of published studies dealing with AP in treating previable rupture of FM is summarized in Table 5. The authors are familiar with the fact that some of the studies listed in Table 5 contain both spontaneous and iatrogenic forms of PPROM. However, they find it reasonable based on the lack of the original papers and the rare occurrence of the diagnosis.

**Table 5:** Characteristics of included studies in a systematic review of amniopatch for sPPROM vs iPPROM—adapted from [40]

Author (year of publication)/origin	Study type	Type of PPROM (sample size)	Mean GA at PPROM diagnosis	GA at amniopatch	GA at delivery	Fluid reaccumulation	Chorioamnionitis	Adverse fetal outcomes
Ferianec et al. [41]/ Slovakia University Hospital Bratislava	Multicentric comparative	sPPROM (53)	19.4	22	27.6	NA	0%	NA
Maged et al. [43]/Minia University Hospital, Egypt	RCT	sPPROM (50)	28	27.5 ± 2.4	34.4	44%	4%	10%
		Expectant (50)	27					
Sung et al. [4]/Samsung Medical Center, South Korea	Retrospective cohort	sPPROM (17)	17	19.3 (17.0–21.7)	22.0 (17.0–40.0)	36.4%	37.5%	Stillbirth 9.1%; IUFD 45.4%
		iPPROM (11)		20.2 ± 2. 4	32.3 ± 4.8	63.2%	10.5%	
Kwak et al. [7]/Samsung Medical Center, Sungkyunkwan School of Medicine, Korea	Case series	sPPROM (7)	21	22.5 (21.1–23.5)	27.6 (21.3–39.0)	14.3%	0	0
Ferianec et al. [42]/Slovakia University Hospital Bratislava	Case series	sPPROM (1)	20	21.2	33.2	100%	0	0
Contino et al. [30]/Maria Vittoria Hospital, Turin, Italy	Case series	sPPROM (2)	21	21.0	27.0	50.0%	NA	NA
		iPPROM (3)						

*sPPROM* spontaneous previable rupture of membranes, *iPPROM* iatrogenic previable rupture of membranes, *IUFD* intrauterine fetal demise, *GA* gestational age, *NA* not applicable

## Non-coagulant components of amniopatch: possible therapeutic effect?

These findings prompted the authors to review the literature in detail on the potential effect of the individual components of AP as the key to understanding the molecular nature of their therapeutic effect [1, 41]. Most studies investigating the plasma cryoprecipitate (Cryo) effect focus principally on the procoagulant effect of its components. Historically, the first published paper by J. P. Allain et al. [44] mentioning the composition of Cryo in the context of its non-coagulant components was made public in the *Scandinavian Journal of Haematology* entitled *Non-Factor VIII related constituents in concentrates*. The approximate composition of Cryo described in the text of this manuscript is summarized in Table 6.

**Table 6:** Overview of the constituents of the conventionally used cryoprecipitate—adapted from [44]

Protein content (%)						
Product	Fibrinogen	Fibronectin	Immunoglobulin M	Immunoglobulin G	Albumin	Total content
Plasma cryoprecipitate	60.4	22	2.4	7.2	6	98

## Isohemagglutinins

Since publishing the above study, the non-coagulant components of Cryo have been dealt with mainly in immunohematology journals in the context of hemotherapy and the risk of developing complications related to incompatibility in the AB0 system of blood groups. A study by Canadian authors (2021) reports an increased incidence of anti-A and anti-B antibodies from the carbohydrate-specific immunoglobulin class (CSA), the so-called isohemagglutinins (IHA), in Cryo-type preparations [45]. The mean concentration of anti-A and anti-B immunoglobulins G (IgG) in Cryo is 6.61 ± 1.4 g/l, while in human plasma (Pm), it is 5.81 ± 1.2 g/l. A statistically significant difference in the above preparations in favour of Cryo was noted for concentrations of anti-A and anti-B immunoglobulins M (IgM) (1.42 ± 0.6 vs 0.49 ± 0.3 g/L, *p* < 0.01).

The association of a different antigenic phenotype of the ABO blood group system [ISBT 001] with an individual’s susceptibility or resistance to specific infections has been known for a long time [46]. Its molecular determinant is the presence of compatible IHA in Pm with their cross-reactivity with some bacterial antigens. The antigens of the ABO system are chemically glycoproteins and glycolipids, while the critical antigenic component of these molecules (epitope) is the glycosylation chain of terminal oligosaccharides. Blood group O corresponds to the presence of l-fucose (Fuc), blood group A the presence of *N*-acetyl-d-galactosamine (GalNAc), and blood group B the presence of d-galactose (Gal) [47]. The biological phenomenon of IHA emergence is associated with colonising the neonatal intestine by a species-specific microbiome in the early postpartum period, with secondary stimulation of mucosal lymphocytes (GALT) by relevant bacterial antigens. The titer of compatible IHAs also changes throughout later life based on quantitative and qualitative changes in the gut microbiota (taking probiotics, antibiotics, and surgical procedures on the colon) [48].

Based on the similarity of microbial antigens, the cross-reactivity of anti-B IHA with bacterial antigens (Gal) *Plasmodium falciparum*, *Escherichia coli*, *Streptococcus agalactiae*, *Staphylococcus aureus* and the family *Enterobacteriaceae* and anti-A IHA with bacterial antigens (GalNAc) *Streptococcus pneumoniae* and *Neisseria gonorrhoeae* has been documented (Table 7) [47].

**Table 7:** Overview of the cross-reactivity of human isohemagglutinins with particular bacterial species—adapted from [47]

Bacterial species	Blood group	Isohemagglutinin present	Clinical outcome
*Escherichia coli*	A	anti-B	Increased susceptibility
*Salmonella* sp.	A	anti-B	Increased susceptibility
*Streptococcus agalactiae*	A	anti-B	Increased susceptibility
*Streptococcus pneumoniae*	B	anti-A	Increased susceptibility
*Staphylococcus aureus*	A	anti-B	Increased resistance
*Neisseria gonorrhoeae*	B	anti-A	Increased resistance

*anti-A* isohemagglutinin type A, *anti-B* isohemagglutinin type B

This phenomenon may be a significant factor in reducing bacterial charge in amniotic fluid in cases of sPPROM treated with AP application. Of the selected bacterial families, *Streptococcus* and *Enterobacteriaceae sp*. are particularly important.

## Plasma fibronectin

Another of Cryo’s non-coagulant components is plasma fibronectin (pFN). Plasma FN is a multidomain, soluble glycoprotein (440 kDa) with the function of potent opsonin, which occurs in the plasma of several vertebrate species, including humans. After pFN binding to corpuscular antigens (microorganisms, tissue fragments), their clearance is provided by the reticuloendothelial system (RES) [49]. The clinical significance of pFN as opsonin has been documented in several patients with sepsis and after severe physical traumas (polytrauma, burns). In such cases, progressive “consumption” occurs, while a reduced serum level is associated with a poor prognosis [50].

Chen et al. [26] described the presence of pFN in amniotic fluid for the first time in 1976 in an article entitled *Identification of the cold-insoluble globulin of plasma in amniotic fluid* published in the *American Journal of Obstetrics and Gynecology*. Afterwards, the dynamics of its concentrations depending on gestational age were described by a Japanese working group led by H. Negishi in 1995 [51]. Regarding the opsonizing function of pFN and its natural occurrence in amniotic fluid, the dynamics of its concentrations in the context of IAI and contractile activity in the study of Romero et al. were investigated. No statistically significant difference was found. The outcome of the study may also have been influenced by the fact that intra-amniotic infection was defined for its needs as the culture capture of microorganisms from the amniotic fluid without determining the presence of inflammatory activity (inflammatory markers in the amniotic fluid). However, this definition does not allow the distinction of intra-amniotic infection from harmless colonization, which is not accompanied by a maternal–fetal inflammatory response. Without an inflammatory response or the initial phase of intra-amniotic infection, the concentration of pFN in the amniotic fluid may be within the physiological range [52]. According to the authors, exogenous, intra-amniotic administration of pFN in the form of AP may be associated with an increased opsonizing capacity of amniotic fluid, leading to a decrease in local microbial charge and the production of proinflammatory cytokines.

## Platelets

Platelets in the platelet concentrate represent a dominant element of primary physiological hemostasis. While this function has been known since its discovery by the Italian physician Bizzozero in 1881, recently, platelets have gained recognition for their no less significant immune, immunomodulatory, and anti-infectious functions [53, 54].

Circulating platelets are a potent component of innate immunity and are involved in the sequestration and eradication of a wide variety of microorganisms, performing this “task” in several ways. Families of cationic proteins from the group of thrombocidines, defensins, and kinocidines are responsible for their direct microbicide effect.

In addition to this effect, platelets on their surface are provided with groups of innate immunity receptors from the Pattern recognition receptors (PRR) group (TLR 4), receptors for complement components, and receptors for immunoglobulins (FcγRIIA), which allow them to bind both native and opsonized bacteria with their possible internalization into the cytoplasmic compartment. Such adhered bacteria are subsequently limited in their natural movement. When platelets aggregate into the form of a thrombus, they concentrate (sequester) in this area, thus limiting their spread and facilitating eradication. The interaction of platelets and neutrophilic leukocytes with their activation and incorporation into the structure of the primary thrombus has been identified as the so-called phenomenon of immunothrombosis. In addition to the concentration of microorganisms and components of innate immunity (neutrophilic leukocytes and platelets) at one site, neutrophilic leukocytes are also activated with their subsequent production of neutrophilic extracellular traps (NETs). NETs are formed by the apoptosis of neutrophilic leukocytes (the so-called NETosis). They are composed of fragments of nucleic acids, base proteins of the nature of histones, myeloperoxidase, elastase and pentraxins, which mediate the elimination of a wide range of pathogens [55, 56].

The above can have two significant implications in the context of the therapeutic effect of AP. The first represents a potentially increased clearance of microorganisms in the amniotic cavity through platelets applied in AP. The second represents the potential “consumption” of the amniotic “pool” of platelets administered in the implementation of AP with their insufficient concentration at the site of the FM defect, which may reduce its effectiveness.

The anti-inflammatory and reparative effect of platelets and plasma derivatives is documented by some preclinical (animal) and clinical studies using plasma-rich platelets in the form of preparations of different viscosity (liquid extracts, extracellular matrices, biopolymers) in the treatment of chronic skin defects, soft tissue defects, bone-muscle structures, and the eye. In this regard, a review study of an Argentinian working group led by J. Etulain, published in 2018 in the journal *Platelets* entitled *Platelets in wound healing and regenerative medicine,* summarizing publishing activity in this field from the early 1990s to 2018, is of great benefit. The listed postulates were also documented in a recent study by Chinese authors of 2022 entitled *Effects of intrauterine infusion of platelet-rich plasma on hormone levels and endometrial receptivity in patients with repeated embryo implantation failure*, investigating the effect of the intrauterine application on plasma-rich platelets in the field of assisted reproduction. Published findings report improved endometrial trophy and increased uterine blood supply after intra-amniotic application of therapeutic hemoderivates [57].

## Discussion

### Amniopatch as a tool for suppression of intra-amniotic inflammation?

When choosing a therapeutic approach and establishing a prognosis for pregnancy complicated by the development of sPPROM, the determination of the presence or absence of IAI and bacteria in the amniotic cavity is significant (Table 2).

The single presence of bacteria in the amniotic fluid without a fetal-maternal inflammatory response may not be associated with a negative perinatal outcome. The intra-amniotic-inflammatory activity of infectious or non-infectious nature can lead to fetal inflammatory response syndrome (FIRS), which interferes with the physiological development of the fetus and functional organ maturation. As a result, it is associated with shortening the latency interval (LP) from detection of sPPROM to childbirth (higher incidence of childbirth up to 48 and 72 h from PPROM detection compared to the absence of FIRS [48 h.: 88% vs 29.7%; 72 h.: 88% vs 35%; *p*-value: <0.05]) and increased rates of neonatal morbidity in the following categories: (1) early neonatal sepsis (RR = 3.1), (2) BPD (RR = 5.9), (3) IVH (RR = 4.9), (4) PVL (RR = 3.3), and (5) RDS (RR = 2.4) compared to its absence. Despite the multi-organ nature of fetal disability, FIRS is predominantly associated with fetal brain damage [52]. These facts are also documented by the current study (2022) of American authors presented in the journal *American Journal of Obstetrics and Gynecology* entitled *Acute histologic chorioamnionitis independently and directly increases the risk for brain abnormalities seen on magnetic resonance imaging in very preterm infants* [58]. At the molecular level, the mechanism of its involvement is attributed to elevated levels of proinflammatory cytokines (interleukin-1 [IL-1], tumour necrosis factor α [TNF-α]) in the amniotic fluid, which lead to an increase in the permeability of the blood–brain barrier. Such altered microvasculature of the white matter of the brain allows for an increased passage of cytokines (especially interleukin 6 [IL-6]), bacteria and their metabolites, which stimulate microglial cells to the increased local production of cytokines. A high concentration of cytokines disrupts the process of primitive myelination and cytotoxicity.

Two important conclusions emerge from the above: (1) Intra-amniotic inflammation and FIRS are associated with shortening pregnancy duration, and (2) Even the effective prolongation of pregnancy duration with simultaneous inflammatory activity in the amniotic cavity is associated with unfavourable perinatal outcomes.

The authors assume that the above components of AP (IHA, pFN, platelets) can effectively alter the intra-amniotic charge of microorganisms and proinflammatory cytokines, thus contributing to the suppression of enzymatic lysis of FM and the reduction of risks associated with IAI and FIRS. In addition to this function, they can be a source of growth factors potentiating reparative processes. The above assumptions arise from the published observations of the authors describing the absence of clinically manifested ChA, with a simultaneously reduced incidence of RDS and ROP in cases of sPPROM treated with AP application. The fact that the above observations could not be explained by a purely physical–mechanical correction of pressure–volume parameters in the amniotic cavity led the authors to look for alternative hypotheses explaining the improvement of perinatal outcomes [1, 41].

In terms of pros and cons of the described technique, based on its nature, it has a similar risk profile of complications as AMC with 0.8% increase in spontaneous miscarriage rate (2.1–1.3%; RR 1.6; 95% CI: 1.02–2.52) so it can be considered relatively safe [59]. The potential of the technique itself in the field of sPPROM treatment has remained unclear until recently, but its possible benefit has been documented by various non-RCT studies. Two of them were published by the authors of the submitted paper: (1) *Amniopatch as an active treatment of spontaneous previable rupture of membranes* (The Journal of Maternal–Fetal & Neonatal Medicine®): this study proved the potential of AP for prolongation of the latency interval between sPPROM and delivery, (2) *Is amniopatch an effective treatment for spontaneous previable premature rupture of membranes? Analysis of perinatal outcome*: this study proved the potential of AP for the reduction of RDS and maternal endometritis in cases of sPPROM [1, 41].

Based on the poor prognosis of sPPROM if left untreated or treated in the conventional manner (expectant management), the authors are convinced about the possible favourable properties of AP in the treatment of the abovementioned diagnosis. The authors maintain that the inclusion of AP in the standard sPPROM therapeutic algorithm for a selected group of patients can streamline the management of such severely affected pregnancies. The essential selection criterion for the AP treatment is the parental request for its application. The other criteria recommended by the authors are summarized in Table 8.

**Table 8:** The inclusion criteria for the AP treatment in cases of sPPROM—adapted from [1]

Number	Used criteria	Clinical definition
1	Singleton pregnancies	Defined by ultrasound
2	sPPROM	sPROM before 24 gw
3	Absence of fresh vaginal bleeding	Defined clinically
4	Latency interval of more than 10 days	Defined clinically
5	The presence of oligohydramnios or anhydramnios	Ultrasound—DVP less than 2 cm
6	The absence of maternal inflammatory markers positivity	No laboratory-defined maternal inflammatory syndrome
7	The absence of severe congenital malformations or chromosomal aberrations	Defined by ultrasound examination or karyotype examination

*sPPROM* spontaneous previable rupture of membranes, *DVP* deepest vertical pocket, *gw* gestational week

### Prospective clinical implications

The paper points to the importance of inflammatory changes in the pathogenesis of sPPROM and a need to subcategorize this syndromological unit (Table 2) based on the presence or absence of bacteria and intra-amniotic inflammation in the amniotic fluid, considering the degree of their severity secondarily. What is significant for clinical practice is the fact that the effect of AP does not have to consist in a highly mechanical, valved closure of the FM defect [42], but in the complementary optimization of the intrauterine environment of the fetus through the reduction of local bacterial and cytokine charge, allowing the development of the organ systems of the fetus. For detailed verification of the given postulate, determining the IL-6 levels and the presence of bacteria in amniotic fluid samples before and after the application of AP is necessary. At the same time, the decreasing dynamics of their concentrations combined with a favourable perinatal outcome constitute confirmatory evidence.

To date, this postulate needs to be supported by sufficiently relevant data. It assumes that the literarily documented bactericidal, opsonizing, immunomodulatory, and reparative effect of the non-coagulant components of AP (IHA, pFN, platelets) is similar in conditions in utero with the simultaneous synergy of their interaction.

In conclusion, it is important to note that there has been a lot of published data (Table 5). However, none of them is in the category of RCT, based on the rare occurrence of the diagnosis and problematic ethical aspects that make the randomization hardly achievable. However, the generally accepted idea is that the topic of the issues of sPPROM needs to be the subject of intensive international research. The authors of the submitted paper already published (2022) the most extensive set of pregnancies complicated by sPPROM treated by AP [41].

## Data Availability

The background for the formulation of the hypothesis on the potential anti-infectious and anti-inflammatory action of non-coagulant components of amniopatch involved: i) clinical-academic and publishing outputs of the authors based on their many years’ experience with amniopatch application in the treatment of spontaneous previable rupture of membranes (2008–2019), ii) the documented absence of clinically manifested chorioamnionitis in patients treated this way with a simultaneously reduced incidence of neonatal respiratory distress syndrome compared to expectant management (tocolysis, corticotherapy, antibiotic therapy). The suporting data can be found in authors previous manuscripts published [1, 41].
